# Tolerance-guided dose adjustment in sublingual immunotherapy for children with dust mite–induced allergic rhinitis: a 6-month prospective cohort study

**DOI:** 10.3389/fmed.2026.1904191

**Published:** 2026-09-10

**Authors:** Meilan Wang, Zhengcai Li, Jing Song, Yang Zhou, Xi Dai, Yao Deng, Jing Ma, Yingqin Gao

**Affiliations:** Department of Otolaryngology-Head and Neck Surgery, Kunming Children’s Hospital, Kunming, China

**Keywords:** allergic rhinitis, children, dose adjustment, individualized treatment, lung function, nasal allergen challenge, nitric oxide, sublingual immunotherapy

## Abstract

**Background:**

Sublingual immunotherapy (SLIT) is effective for allergic rhinitis (AR), though its efficacy exhibits inter-individual variability and is dose-dependent. Standardized protocols may not achieve optimal outcomes in all pediatric patients, highlighting the need for individualized dose-adjustment strategies. The nasal allergen challenge (NAC), the gold standard for assessing nasal mucosal reactivity, provides an objective tool to evaluate treatment response and guide dose personalization.

**Objective:**

To evaluate the short-term (6-month) efficacy of individualized SLIT dose adjustment in children with dust mite–induced AR, and to assess its effects on upper and lower airway inflammation and lung function.

**Methods:**

In this prospective cohort study, 235 children (3–14 years) with dust mite–induced AR were assigned to a standard-dose group (SD, *n* = 133) or a dose-adjustment group (DA, *n* = 102). The DA group received an incrementally increased maintenance dose based on tolerance (up to 7 drops of potency No. 4). Outcomes were assessed at baseline and after 6 months, including symptom and medication scores (TNSS, TMS, CSMS, VAS, RQLQ), NAC reactivity, lung function (FEV₁ Z-score), fractional exhaled nitric oxide (FeNO), and nasal nitric oxide (nNO). Baseline characteristics were generally balanced between groups, except for age and disease duration, which were adjusted for in multivariable analyses.

**Results:**

After 6 months, all symptom scores improved significantly in both groups (all *P* < 0.001), with greater improvement in the DA group after adjusting for age and disease duration (TNSS: B = −0.648, 95% CI: −1.186 to −0.109, *P* = 0.019; TMS: B = −0.141, 95% CI: −0.184 to −0.098, *P* < 0.001; CSMS: B = −0.304, 95% CI: −0.448 to −0.161, *P* < 0.001; VAS: B = −0.893, 95% CI: −1.295 to −0.490, *P* < 0.001; RQLQ: B = −1.282, 95% CI: −2.152 to −0.413, *P* = 0.004). A higher proportion of patients in the DA group showed reduced NAC reactivity (adjusted OR = 1.67, 95% CI: 1.01–2.75, *P* = 0.046). Both groups exhibited significant reductions in FeNO and nNO (all *P* < 0.05). The DA group also showed a significant increase in FEV₁ Z-score from baseline (mean change = 0.725, 95% CI: 0.299–1.150, d = 0.822, *P* = 0.002), and the value was significantly higher than that in the SD group (mean difference = 0.872, 95% CI: 0.138–1.605, d = 0.714, *P* = 0.021).

**Conclusion:**

In this prospective pediatric cohort, individualized SLIT dose adjustment based on tolerance was associated with better short-term outcomes, reduced nasal mucosal reactivity, and improved lung function. These findings support the feasibility of tolerance-guided dose personalization and warrant confirmation in randomized controlled trials.

## 1 Introduction

Allergic rhinitis (AR) is a common inflammatory upper airway disease that imposes a significant health burden worldwide. In China, Epidemiological studies indicate a high and rising prevalence of AR, particularly among children, with a diagnosis rate ranging from 10.8% to 21.09% (1–3). Dust mites are recognized as the most prevalent inhalant allergen triggering AR in children (4, 5). Rapid urbanization and environmental changes are expected to further drive this upward trend in AR prevalence (6). Sublingual immunotherapy (SLIT) is a well-established, disease-modifying treatment for allergic diseases (7, 8). In particular, house dust mite SLIT, which has been used in China for nearly two decades, has demonstrated confirmed efficacy and safety in treating AR (7, 9). However, the efficacy of SLIT varies among individuals, and existing evidence indicates that clinical outcomes are closely related to both the maintenance dosage and adherence (10). Therefore, personalized dosage adjustment may be a key factor in optimizing efficacy. Clinical practice suggests that around 6 months of immunotherapy represents a critical window for evaluating early efficacy. During this period, patients typically begin to show symptomatic improvement, which can verify the effectiveness of treatment. Additionally, early benefits can enhance patient confidence and significantly improve long-term adherence (11–13).

Currently, the evaluation of SLIT efficacy relies primarily on subjective indicators, whereas objective tools remain underutilized. The nasal allergen challenge (NAC) is the gold standard for diagnosing AR, enabling precise identification of key allergens and simulating the natural disease process (14, 15). Moreover, NAC has demonstrated considerable value as an objective endpoint for assessing therapeutic efficacy in AR. For instance, a clinical trial in Spain on AR desensitization therapy employed NAC as a primary efficacy indicator to evaluate improvements before and after treatment (16). However, in China, there are no studies utilizing NAC for evaluating the efficacy of desensitization therapy. Nitric oxide (NO) is a crucial regulator of airway inflammation. Fractional exhaled nitric oxide (FeNO) primarily reflects inflammation in the lower airways (bronchi), and an elevation in its levels is often regarded as an indicator of increased risk for lower airway inflammation or increased asthma risk in patients with AR (17, 18). Nasal nitric oxide (nNO) mainly assesses the patency and inflammatory status of the upper airways (nasal cavity and sinuses), and the control of nasal inflammation can be reflected through its dynamic changes (19).

AR has been closely linked to pediatric asthma, with a significantly higher risk of asthma development demonstrated in children affected by AR (18). Therefore, pulmonary function testing in AR children is essential. SLIT can alleviate the inflammation in both the upper and lower airways, and modulate the interaction between nasal and bronchial inflammation. Current research indicates that house dust mite SLIT improved pulmonary function parameters and reduced FeNO levels in children with allergic diseases, yet its impact on nNO levels remains insufficiently studied (20).

## 2 Materials and methods

### 2.1 Ethical statement

This study protocol was approved by the Medical Ethics Committee of Kunming Children's Hospital (approval number: 2023-03-383-K01). Written informed consent was obtained from all patients and their legal guardians after they were fully informed of the study purpose and procedures.

### 2.2 Patients

This prospective study was conducted in the Department of Otolaryngology-Head and Neck Surgery at Kunming Children's Hospital from April 2024 to August 2025, involving a total of 250 pediatric participants aged 3–14 years, with age selection based on the Chinese guideline (21). Inclusion criteria were as follows: (1) diagnosis of intermittent or persistent AR according to the Allergic Rhinitis and its Impact on Asthma guidelines; (2) a history of sensitization to *Dermatophagoides farinae* and/or *Dermatophagoides pteronyssinus*, confirmed by specific immunoglobulin E (sIgE) levels ≥0.7 kU/L or positive skin prick test (SPT), defined as a mean wheal diameter >3 mm greater than the negative control. An additional enrollment requirement of skin index (SI) ≥ 0.5 (i.e., ‘++’ or above) was applied; this SI was used only for grading reaction intensity and screening, not for defining sensitization; (3) positive NAC to *Dermatophagoides farinae* allergen extract. Exclusion criteria included severe or uncontrolled asthma, major systemic diseases, and any condition that would preclude adequate cooperation or completion of the NAC, such as inability to understand instructions or tolerate the procedure.

### 2.3 Study design

Patients were treated with *Dermatophagoides farinae* drops (Zhejiang Wolwo Bio-pharmaceutical Co., Ltd., China). The drops were labeled from No. 1 to No. 5 with increasing total protein concentrations of 1, 10, 100, 333, and 1,000 μg/mL, respectively. Patients under 14 years of age used No. 1 to No. 4. The drops were administered sublingually for 1–3 min before swallowing. Each drop was approximately 0.05 mL, and no food or drink was allowed within 15 min after administration. The first dose was administered in the hospital under 30-minute observation.

**Figure 1:**
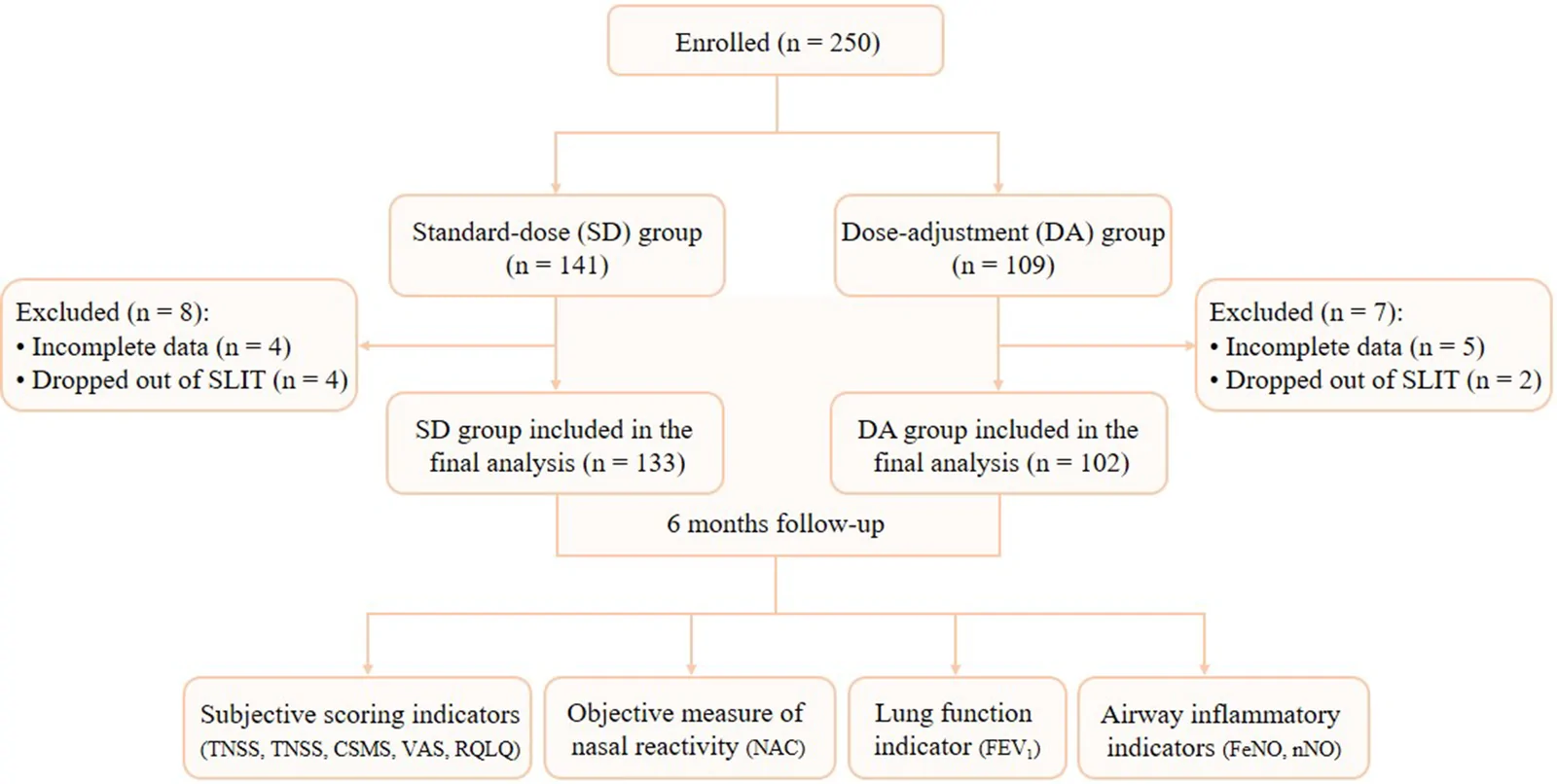
Study flow chart.

The 250 eligible pediatric patients were allocated to the standard-dose (SD) group or the dose-adjustment (DA) group through shared decision-making between physicians and patients/guardians after thorough counseling on the known risks and potential benefits. Allocation considered both clinical recommendations (including tolerance assessment and safety considerations) and patient/guardian preference. The study flow chart is shown in Figure 1.

Both groups received SLIT with *Dermatophagoides farinae* drops and took symptomatic medications as needed. The SD group followed a standardized dosing regimen (Table 1). The dose-adjustment strategy in the DA group drew on prior evidence supporting stepwise up-titration (22, 23). Patients in the DA group attempted to escalate the dose starting from the fourth week of treatment, beginning with 3 drops of No.4, with weekly increments of 1 drop, up to a maximum of 7 drops daily (Table 2). If intolerance (oral numbness or pruritus, fatigue, gastrointestinal reactions, and local rashes) occurred during dose adjustment, the dose was reduced by one level, which then served as the final maintenance dose.

**Table 1:** Dosing regimen for the SD group.

Specification	Time	Day 1	Day 2	Day 3	Day 4	Day 5	Day 6	Day 7
No. 1	First week	1 Drop	2 Drops	3 Drops	4 Drops	6 Drops	8 Drops	10 Drops
No. 2	Second week	1 Drop	2 Drops	3 Drops	4 Drops	6 Drops	8 Drops	10 Drops
No. 3	Third week	1 Drop	2 Drops	3 Drops	4 Drops	6 Drops	8 Drops	10 Drops
No. 4	Fourth week	Once daily, 3 drops each time.						
Beginning from the fourth week, administer No. 4 once daily, 3 drops each time.								

**Table 2:** Dosing regimen for the DA group.

Specification	Time	Day 1	Day 2	Day 3	Day 4	Day 5	Day 6	Day 7
No. 1	First week	1 Drop	2 Drops	3 Drops	4 Drops	6 Drops	8 Drops	10 Drops
No. 2	Second week	1 Drop	2 Drops	3 Drops	4 Drops	6 Drops	8 Drops	10 Drops
No. 3	Third week	1 Drop	2 Drops	3 Drops	4 Drops	6 Drops	8 Drops	10 Drops
No. 4	Fourth week^a^	Once daily, 3 drops each time.						
No. 4	Fifth week^a^	Once daily, 4 drops each time.						
No. 4	Sixth week^a^	Once daily, 5 drops each time.						
No. 4	Seventh week^a^	Once daily, 6 drops each time.						
No. 4	Eighth week^a^	Once daily, 7 drops each time.						

a This phase is for dose adjustment. Concomitant use of symptomatic medications is withheld during this time to ascertain the maintenance dose. The final maintenance dose is determined by de-escalating one dose level from the dose at which the patient first exhibits an intolerance reaction during the adjustment period.

### 2.4 Follow-up and assessments

This study included telephone follow-ups and on-site visits, as detailed below: All enrolled patients were required to undergo on-site visits during the screening period, the run-in period, and after 6 months of treatment to assess the short-term efficacy and safety of SLIT with dust mite extracts. Additionally, telephone follow-ups were conducted by the investigators every 2 months to record safety data.

Screening period (V1): Patients reviewed recent allergic symptoms and medication use. Investigators recorded baseline subjective symptom scores, including the total nasal symptom score (TNSS), medication score (TMS), visual analog scale (VAS) score, and rhinoconjunctivitis quality of life questionnaire (RQLQ) score. Among these, TNSS and TMS were used to calculate the combined symptom and medication score (CSMS).

Run-in period (V2): Conducted during an asymptomatic period prior to therapy initiation. Assessments included FeNO, nNO, forced expiratory volume in one second (FEV_1_) and nasal allergen challenge (NAC) to determine the threshold allergen concentration eliciting a positive response. Additionally, during this on-site visit, physicians were required to prescribe *Dermatophagoides farinae* drops and symptomatic medications to the patients.

6-month follow-up (V3): Conducted during an asymptomatic period after 6 months of therapy. For subjective assessment, patients reviewed their recent TNSS, TMS, VAS, and RQLQ scores; these were recorded by investigators, and the CSMS was derived from the TNSS and TMS. For objective assessment, patients underwent the same battery of tests as in V2, including FeNO, nNO, FEV_1_, and NAC.

### 2.5 Evaluation indicators

#### 2.5.1 Subjective symptom scores

TNSS: the sum of scores for four nasal symptoms (itching, congestion, sneezing, rhinorrhea), with each symptom scored from 0 to 3 (0 = no symptoms, 3 = severe symptoms), resulting in a total score range of 0 to 12.

TMS: scored as 0 (no medication), 1 (oral antihistamines and/or leukotriene receptor antagonists only), 2 (intranasal corticosteroids), or 3 (oral corticosteroids).

CSMS: calculated as CSMS = (TNSS/4) + TMS.

VAS: patients marked a 0–10 cm line to indicate symptom severity, where 0 represented no symptoms and 10 represented the most severe symptoms.

RQLQ: the pediatric rhinoconjunctivitis quality of life questionnaire was used, with higher scores indicating worse quality of life (24). For children aged 3–5 years, trained interviewers read the items aloud, with parents only clarifying wording when necessary; for those aged 12–14 years, the same questionnaire was retained to ensure data consistency (25).

#### 2.5.2 Nasal allergen challenge

The NAC protocol began with a negative-control challenge using phosphate-buffered saline (PBS) to exclude nasal hyper-reactivity. TNSS and nasal resistance were measured before and after PBS administration. Subsequently, bilateral NAC was performed with one spray (100 μL/spray) of *Dermatophagoides farinae* extract. TNSS and nasal resistance were reassessed 15 min after allergen provocation. Allergen concentrations were applied in ascending order (50, 500, 5,000 *μ*g/mL) until a positive response occurred or the highest concentration was reached.

A positive NAC was defined as meeting any of the following criteria (14, 26):

1) An increase in TNSS of 3–4 points and a ≥ 20% decrease in nasal airflow at 150 Pa;
2) An increase in TNSS of ≥5 points;
3) A ≥ 40% decrease in nasal airflow at 150 Pa.

The lower the allergen concentration that induces a positive NAC result, the higher the nasal mucosal reactivity (26). In our study, if the allergen concentration required for a positive NAC increases or the NAC result turns negative after six months of treatment, it indicates a decrease in nasal mucosal reactivity. Conversely, if the allergen concentration required for a positive NAC decreases after six months of treatment, it indicates an increase in nasal mucosal reactivity.

#### 2.5.3 Lung function and nitric oxide measurements

All participants were aged ≥6 years and were able to cooperate with the FEV₁, FeNO, and nNO measurements described below.

FEV_1_: Measured with a portable electronic spirometer (BH-AX-MAPG, Guangzhou Hongxiang Medical Technology Co., Ltd., China). Before testing, children were trained through a candle-blowing game. During measurement, subjects sat upright with a nose clip; after several tidal breaths, they inhaled deeply and then exhaled forcefully with an effort duration of ≥6 s. The maneuver was repeated three times with at least 1-min intervals between attempts. The device automatically rejected unacceptable curves and repeated the procedure until three acceptable curves were obtained. The highest FEV₁ value was recorded as Z-scores derived from the spirometer's built-in reference equations (based on age, sex, height, and weight), with normal defined as Z-score > −1.645 (27).

FeNO: Measured using an online electrochemical analyzer (A101, Zhejiang EnMiles Intelligent Digital Medical Co., Ltd., China) according to the single-breath technique at 50 mL/s. Subjects exhaled with real-time visual feedback, and the plateau FeNO value (ppb) from a valid exhalation was recorded.

nNO: Assessed with the same analyzer fitted with a disposable nasal olive adapter. With one nostril occluded by the probe, subjects held their breath after deep inhalation while the instrument sampled air from the contralateral nostril at a constant flow. The plateau nNO concentration (ppb) was recorded.

### 2.6 Safety assessment

Patients were required to record and report to the investigators the onset time, duration, and symptoms of any adverse events (AEs). Investigators assessed safety based on the incidence and severity of AEs and provided appropriate treatment recommendations promptly.

### 2.7 Statistical analysis

Data were analyzed using SPSS version 26.0 (IBM Corp., Armonk, NY, USA). Normality was assessed via the Shapiro–Wilk test. Baseline characteristics are presented as mean ± SD. For outcome analyses, normally distributed data are reported as mean change with 95% CI, whereas non-normally distributed data are reported as median change with 95% CI derived from the Hodges–Lehmann method. Between-group comparisons were performed using independent-samples t-tests or Mann–Whitney U tests, and within-group comparisons using paired t-tests or Wilcoxon signed-rank tests, as appropriate. Effect sizes for within-group comparisons were calculated as Cohen's d for normally distributed data and as r for non-normally distributed data.

To control for baseline imbalances that could affect treatment effect estimation, group assignment, age, and disease duration were entered as covariates in the regression models (age and disease duration differed significantly between groups at baseline, whereas other baseline variables were balanced and thus not included). Collinearity diagnostics showed that all variance inflation factors (VIF) were < 2, indicating no significant multicollinearity. For continuous outcomes, results from linear regression models are presented as adjusted mean differences (unstandardized regression coefficients B, with 95% CI) comparing the DA group vs. the SD group in change scores (6 months minus baseline); negative B values favor the DA group, indicating greater improvement in the DA group than in the SD group. For the ordinal NAC outcome (coded as 1 = decreased, 2 = unchanged, and 3 = increased reactivity, with higher values indicating worse outcomes), ordinal logistic regression was performed with the DA group as the reference. Accordingly, a common odds ratio > 1 for the SD group vs. the DA group favors the DA group in terms of reducing nasal mucosal reactivity. The proportional odds assumption was verified by the parallel lines test. Risk differences for adverse events and their 95% CIs were calculated using the Wald method. A two-sided *P* < 0.05 was considered statistically significant.

## 3 Results

### 3.1 Patient characteristics

Of the 250 enrolled participants, 235 (94.0%) completed 6 months of treatment with complete follow-up data and were included in the final analysis, including 133 patients in the SD group and 102 patients in the DA group (Figure 1). As shown in Table 3, baseline characteristics were generally comparable between groups in sex, family history of allergy, and baseline scores (TNSS, TMS, CSMS, VAS, RQLQ). However, patients in the SD group had a significantly lower mean age and a shorter disease duration than those in the DA group (all *P* < 0.05). Regarding age distribution, the SD group included 20 children (15.0%) aged 3–4 years, while the DA group contained only 2 children (2.0%) in this age range. Age and disease duration were closely correlated, with older age generally reflecting a longer disease course. Accordingly, the lower mean age in the SD group corresponded to its shorter overall disease duration. This younger age profile in the SD group may be explained by common clinical practice, in which both parents and clinicians exercise greater caution when adjusting medication doses in younger children.

**Table 3:** Baseline characteristics of the study population (*N* = 235).

	SD group (*n* = 133)	DA group (*n* = 102)	*P* values
Age, mean ± SD			
Mean, years	7.32 ± 2.61	8.01 ± 2.20	0.030
Age, n (%)			
3–4	20 (15.0%)	2 (2.0%)	0.001
5–6	36 (27.1%)	24 (23.5%)	
7–14	77 (57.9%)	76 (74.5%)	
Gender, n (%)			
Male	81 (60.9%)	71 (69.6%)	0.166
Female	52 (39.1%)	31 (30.4%)	
Course, mean ± SD			
Mean, years	1.86 ± 1.71	2.45 ± 1.97	0.018
Family history of allergy, n (%)			
Yes	77 (57.9%)	60 (58.8%)	0.886
No	56 (42.1%)	42 (41.2%)	
Baseline data scores, mean ± SD			
TNSS	5.60 ± 1.59	5.71 ± 1.60	0.619
TMS	2.00 ± 0.00	2.00 ± 0.00	1.000
CSMS	3.40 ± 0.40	3.43 ± 0.40	0.619
VAS	4.42 ± 1.05	4.64 ± 1.29	0.169
RQLQ	16.45 ± 5.35	16.94 ± 6.05	0.512

Data are presented as n or mean ± SD as appropriate. *P* values indicate significant differences between the SD group and the DA group.

### 3.2 Clinical efficacy

#### 3.2.1 Assessment of subjective symptom scores

**Figure 2:**
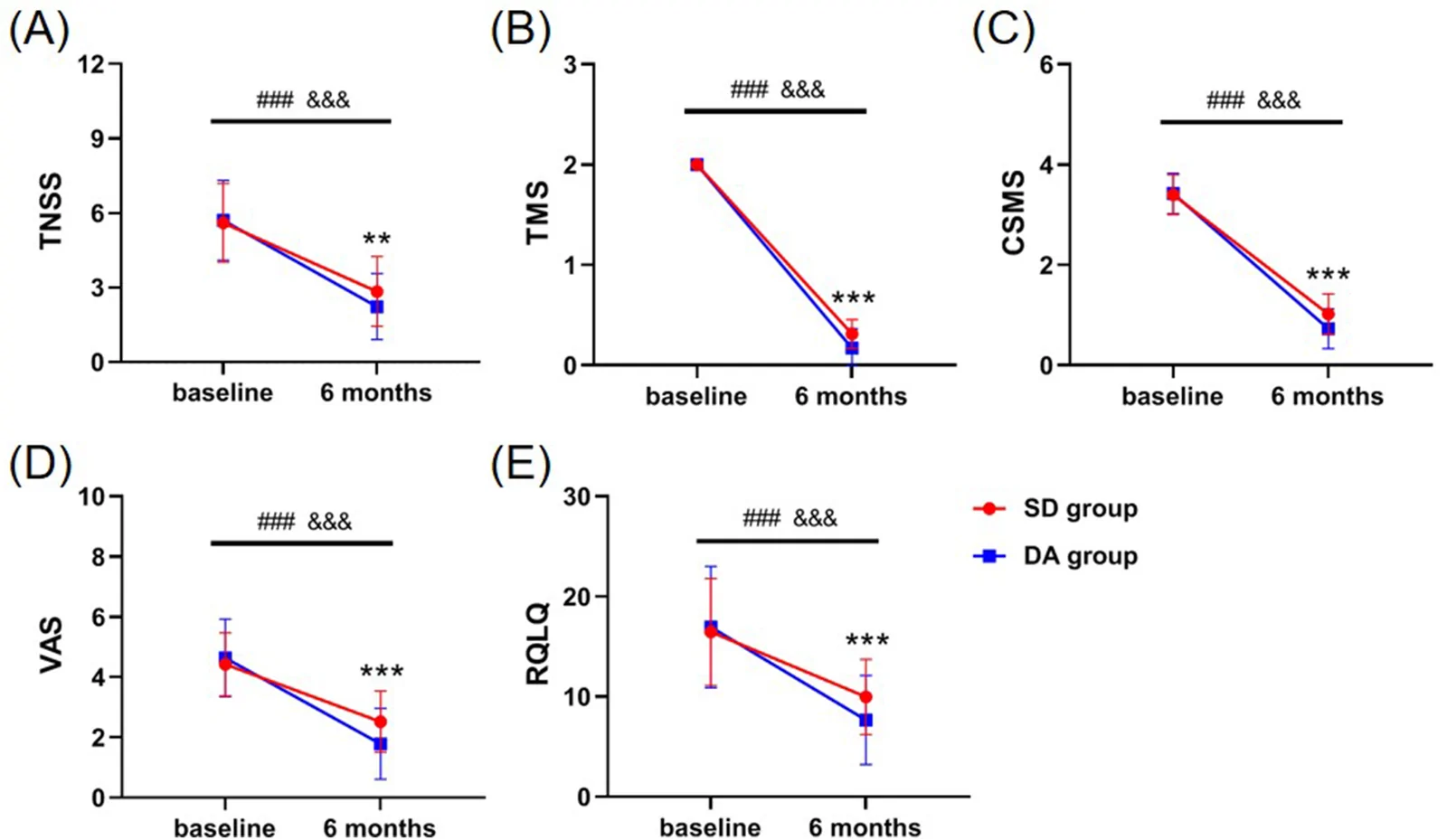
Comparison of efficacy and quality of life between the SD and DA groups. **(A)** TNSS; **(B)** TMS; **(C)** CSMS; **(D)** VAS; **(E)** RQLQ scores. * indicates the difference between the SD group and the DA group **P < 0.01, ***P < 0.001. ^#^ indicates within-group difference in the SD group between baseline and 6-month treatment, ^###^ *P* < 0.001; ^&^ indicates within-group difference in the DA group between baseline and 6-month treatment, ^&&&^ *P* < 0.001.

Following 6 months of treatment, all evaluated scores (TNSS, TMS, CSMS, VAS, RQLQ) were significantly reduced from baseline in both groups (all *P* < 0.001). In the SD group, the median changes (95% CI) were −3.000 (−3.000 to −2.000) for TNSS (effect size r = 0.798), −1.643 (−1.714 to −1.643) for TMS (r = 0.885), −2.393 (−2.464 to −2.286) for CSMS (r = 0.868), −2.000 (−2.000 to −1.500) for VAS (r = 0.804), and −3.000 (−3.500 to −2.500) for RQLQ (r = 0.752). In the DA group, the corresponding median changes (95% CI) were −3.500 (−4.000 to −3.000) for TNSS (r = 0.836), −1.786 (−1.857 to −1.786) for TMS (r = 0.887), −2.696 (−2.804 to −2.571) for CSMS (r = 0.869), −3.000 (−3.000 to −2.500) for VAS (r = 0.836), and −4.500 (−5.000 to −4.000) for RQLQ (r = 0.803). Although no intergroup differences were observed in baseline subjective symptom scores, the DA group showed superior efficacy after treatment, with significantly lower scores than the SD group in: TNSS (median difference = −1.000, 95% CI: −1.000 to 0.000, r = 0.197, *P* = 0.003), TMS (median difference = −0.143, 95% CI: −0.286 to −0.143, r = 0.330, *P* < 0.001), CSMS (median difference = −0.286, 95% CI: −0.393 to −0.179, r = 0.370, *P* < 0.001), VAS (median difference = −1.000, 95% CI: −1.000 to 0.000, r = 0.303, *P* < 0.001), and RQLQ (median difference = −1.000, 95% CI: −2.000 to −1.000, r = 0.269, *P* < 0.001) (Figure 2).

Given the significant baseline differences in age and disease duration between groups, multivariable linear regression analyses were performed on the change scores (6 months minus baseline) of all subjective outcomes. After adjustment for age and disease duration, the DA group continued to show significantly greater improvements than the SD group in all subjective measures: TNSS (B = −0.648, 95% CI: −1.186 to −0.109, *P* = 0.019), TMS (B = −0.141, 95% CI: −0.184 to −0.098, *P* < 0.001), CSMS (B = −0.304, 95% CI: −0.448 to −0.161, *P* < 0.001), VAS (B = −0.893, 95% CI: −1.295 to −0.490, *P* < 0.001), and RQLQ (B = −1.282, 95% CI: −2.152 to −0.413, *P* = 0.004), indicating that the superior efficacy of the DA group remained robust after controlling for potential baseline confounders, including age and disease duration.

#### 3.2.2 Comparison of treatment outcomes in nasal allergen challenge

**Figure 3:**
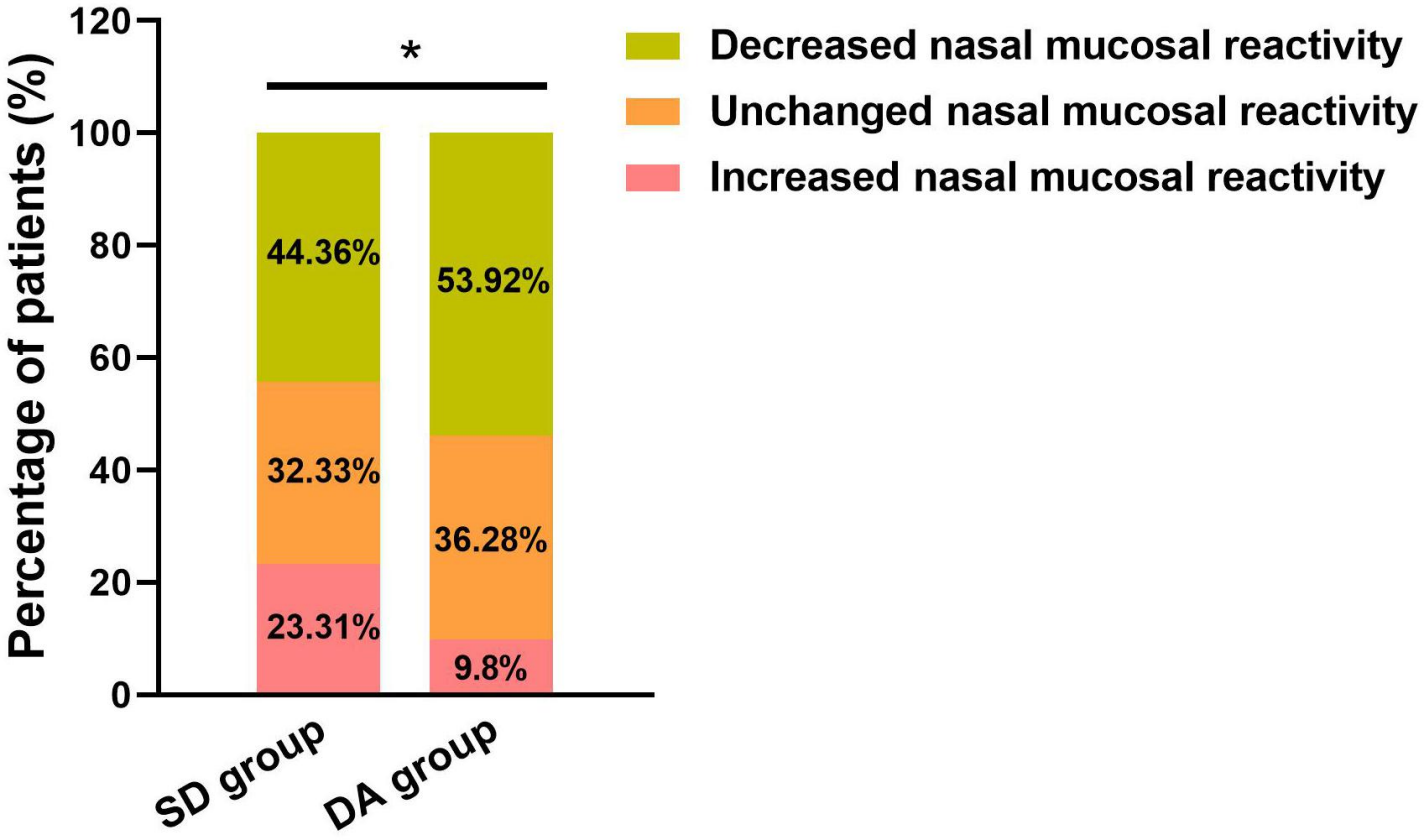
Percentage distribution of nasal mucosal reactivity changes in the SD and DA groups after 6 months of treatment. * indicates the difference between the SD group and the DA group *P < 0.05.

A positive NAC at a lower allergen concentration indicates higher nasal mucosal reactivity and a greater degree of sensitization to the allergen (26). Changes in NAC results for both patient groups after 6 months of treatment are shown in Figure 3. In the SD group, 31 (23.31%), 43 (32.33%), and 59 (44.36%) patients exhibited increased, unchanged, and decreased nasal mucosal reactivity, respectively. In the DA group, the corresponding numbers were 10 (9.8%), 37 (36.28%), and 55 (53.92%) patients. Ordinal logistic regression, adjusted for age and disease duration, showed that the SD group had a significantly higher likelihood of increased nasal mucosal reactivity than the DA group (OR = 1.67, 95% CI: 1.01 to 2.75, *P* = 0.046), indicating a favorable outcome for the DA group in reducing nasal reactivity. The proportional odds assumption was verified by the parallel lines test (*P* = 0.218).

#### 3.2.3 Changes in lung function and inflammatory indicators

To investigate the improvement in patients' lung function and inflammatory indicators, a total of 29 patients in the SD group and 22 patients in the DA group underwent testing for FEV₁ Z-score, FeNO, and nNO. As shown in Table 4, baseline characteristics were well balanced between the two groups in this subset (all *P* > 0.05).

**Table 4:** Baseline characteristics of the subset undergoing lung function and nitric oxide measurements (*n* = 51).

	SD group (*n* = 29)	DA group (*n* = 22)	*P* values
Age, mean ± SD			
Mean, years	8.93 ± 2.61	8.00 ± 1.75	0.122
Gender, n (%)			
Male	20 (69.0%)	15 (68.2%)	0.952
Female	9 (31.0%)	7 (31.8%)	
Course, mean ± SD			
Mean, years	2.29 ± 1.66	2.80 ± 1.79	0.320
Family history of allergy, n (%)			
Yes	11 (37.9%)	15 (68.2%)	0.133
No	18 (62.1%)	7 (31.8%)	
Baseline data scores, mean ± SD			
TNSS	6.07 ± 1.28	6.32 ± 1.13	0.428
TMS	2.00 ± 0.00	2.00 ± 0.00	1.000
CSMS	3.52 ± 0.32	3.58 ± 0.28	0.428
VAS	4.48 ± 0.95	4.86 ± 0.83	0.122
RQLQ	15.86 ± 4.31	17.00 ± 5.04	0.390
FEV_1_ Z-score	−0.67 ± 1.14	−0.29 ± 1.07	0.255
FeNO	5.44 ± 7.64	5.07 ± 5.87	0.704
nNO	644.86 ± 383.50	702.41 ± 381.98	0.597

Data are presented as n or mean ± SD as appropriate. *P* values indicate significant differences between the SD group and the DA group.

**Figure 4:**
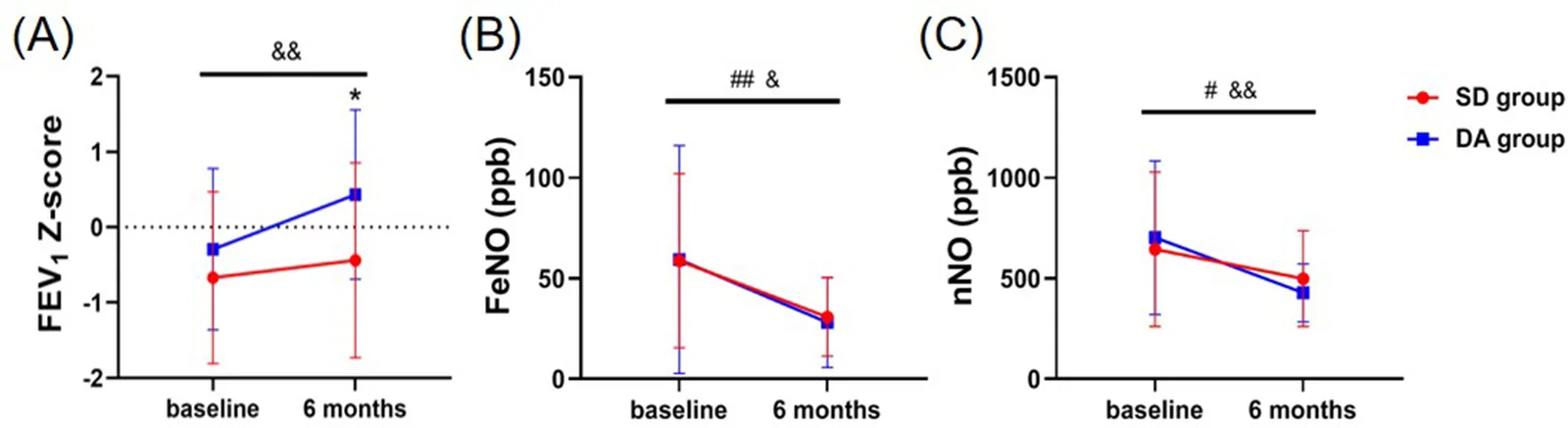
Comparison of FEV_1_ Z-score, FeNO and nNO levels between the SD and DA groups. **(A)** FEV_1_ Z-score; **(B)** FeNO; **(C)** nNO. * indicates the difference between the SD group and the DA group **P < 0.05. ^#^ indicates within-group difference in the SD group between baseline and 6-month treatment, ^#^ *P* < 0.05, ^##^ *P* < 0.01; ^&^ indicates within-group difference in the DA group between baseline and 6-month treatment, ^&^ *P* < 0.05, ^&&^ *P* < 0.01.

Following 6 months of treatment, both groups exhibited an increase in FEV_1_ Z-score from baseline values. Nevertheless, the DA group demonstrated a significant increase (mean change = 0.725, 95% CI: 0.299 to 1.150, d = 0.822, *P* = 0.002). Furthermore, the FEV₁ Z-score was significantly higher in the DA group than in the SD group at 6 months (mean difference = 0.872, 95% CI: 0.138 to 1.605, d = 0.714, *P* = 0.021), whereas no significant difference was observed at baseline (Figure 4A).

Regarding inflammatory indicators, after 6 months of treatment, both the SD and DA groups showed significant reductions from baseline in nNO (SD: mean change = −145.75, 95% CI: −274.44 to −17.07, d = 0.43, *P* = 0.028; DA: mean change = −274.29, 95% CI: −453.20 to −95.38, d = 0.68, *P* = 0.004) and FeNO levels (SD: median change = −14.80, 95% CI: −48.00 to −7.60, r = 0.512, *P* = 0.006; DA: median change = −24.05, 95% CI: −47.75 to −4.23, r = 0.557, *P* = 0.009). However, no significant differences in either nNO or FeNO levels were found between the two groups at baseline or after treatment (Figures 4B and C).

### 3.3 AEs

The incidence of adverse events was 6.77% (9/133) in the SD group and 11.76% (12/102) in the DA group, with a risk difference of −4.99% (95% CI: −12.6% to 2.6%), indicating no statistically significant difference between the two groups (*P* > 0.05). All reported adverse events were mild and local. The majority resolved spontaneously within one week without any intervention, and the remainder were managed with symptomatic medication. In the DA group, 9 of the 12 patients who experienced adverse events during up-titration were reduced by one level per protocol, yielding final maintenance doses of 6 drops (*n* = 4), 5 drops (*n* = 3), and 4 drops (*n* = 2), and all of these patients achieved symptom resolution after dose reduction. The remaining 3 patients developed adverse events during the maintenance phase; symptoms were effectively controlled with symptomatic medication, allowing continuation at the original maintenance doses. No severe adverse events or treatment withdrawals occurred in either group.

## 4 Discussion

Sublingual immunotherapy is a disease-modifying therapy for allergic rhinitis that alters the natural disease course through continuous allergen exposure (7, 8). A substantial body of evidence indicates that the efficacy of SLIT exhibits a time-dependent pattern, with early clinical benefits typically observable within 3 to 6 months after initiation (11, 12). The underlying mechanism involves SLIT modulating immune responses and reducing target organ sensitivity, primarily through the induction of regulatory T cells and the production of allergen-specific IgG4 (28, 29). However, individual variations in patients' immune systems may lead to differential therapeutic responses to standardized SLIT dosing protocols (30). In France, 61% of physicians associate dose escalation with improved efficacy (31). Most clinical studies on house dust mite SLIT in China employ fixed standardized dosing regimens. While such conventional fixed-dose protocols are safe and widely applicable, they may not fully realize the therapeutic potential in some pediatric patients. For example, our previous study demonstrated that patients with a suboptimal response to conventional-dose house dust mite SLIT (with a CSMS improvement rate between 20% and 50%) could achieve CSMS improvement comparable to that of high responders by increasing the dose (22). These observations highlight the need to complement trial data with real-world evidence to guide individualized treatment. As highlighted by Catamerò et al. in a comprehensive evaluation of AIT for allergic rhinitis, integrating findings from both RCTs and real-world studies is essential for informing clinical guidelines (32). Similarly, Paoletti et al. emphasized the value of well-designed observational studies in providing complementary evidence to RCTs (33). Against this backdrop, our prospective cohort study provides real-world data on the feasibility and short-term outcomes of a tolerance-guided dose adjustment strategy in pediatric patients.

In this study, we implemented a tolerance-guided protocol with incremental dose escalation up to a maximum of 7 drops of formulation No. 4 daily (approximately 2.3-fold the standard maintenance dose). After 6 months of treatment, the DA group showed significantly greater improvements in all subjective evaluation metrics (TNSS, TMS, CSMS, VAS, and RQLQ) compared to the SD group, along with a favorable safety profile. Although the incidence of adverse events was slightly higher in the DA group (11.76% vs. 6.77%), the difference was not statistically significant, and all events were mild and manageable local reactions. These findings collectively suggest that for children with dust mite-induced allergic rhinitis, implementing an individualized dose-escalation strategy under close supervision may contribute to symptom control, reduce reliance on symptomatic medications, and improve quality of life in the short term while maintaining safety, and add to the body of evidence regarding the individualized use of SLIT in clinical practice. The observed baseline differences in age and disease duration between groups likely reflect real-world clinical decision-making where more proactive strategies are often reserved for older children with more refractory conditions (34, 35). To mitigate potential confounding by these imbalances, we performed multivariable linear regression analyses adjusting for age and disease duration in all between-group comparisons. After adjustment, the DA group maintained significantly greater improvements than the SD group across all subjective measures. The consistency between unadjusted and adjusted analyses supports the robustness of the between-group differences and suggests that the observed superiority of the DA group is unlikely to be fully attributable to these baseline imbalances.

In the evaluation of AR treatment efficacy, subjective indicators, such as CSMS, VAS, and RQLQ, directly reflect patients' perceptions and functional status, serving as the core of clinical efficacy interpretation (36). Meanwhile, NAC can directly and objectively quantify the sensitivity of the nasal mucosa. Internationally, some studies have utilized NAC as an alternative observational endpoint for assessing the efficacy of AR desensitization therapy (16). However, an objective assessment system in this field remains underdeveloped in China. One feature of this study is the incorporation of the standardized NAC as an auxiliary objective tool for evaluating the efficacy of SLIT for house dust mite allergy in children. The results showed that the DA group had both greater improvements in subjective symptoms and a higher proportion of patients achieving reduction in nasal mucosal reactivity. After adjusting for age and disease duration, the DA group remained associated with a lower likelihood of increased nasal reactivity. These findings suggest that the symptomatic relief observed in the DA group was not only reflected in patient-reported outcomes but was further substantiated by objective evidence, namely a quantifiable decrease in the reactivity of the nasal mucosa to allergens. The congruence between objective and subjective findings lends support to the dose-adjustment strategy, particularly regarding its short-term physiological impact. Nevertheless, NAC effectively captures short-term nasal reactivity changes but is not a standalone surrogate for long-term immune tolerance or disease modification, which would require further longitudinal validation. With this balanced interpretation, our integrated assessment framework, combining subjective scales with an objective physiological test, offers a more comprehensive approach to evaluating SLIT efficacy.

The concept of “one airway, one disease” highlights the close association between upper and lower airway inflammation, with AR being a significant risk factor for asthma (18). As an exploratory adjunct to the primary symptom-based outcomes, this study monitored FEV_1_ Z-score to assess short-term effects of SLIT on lower airway function. Notably, although baseline lung function in both groups of children was within the normal range, the DA group showed a significant increase in FEV₁ Z-score from baseline after treatment, and the FEV₁ Z-score was significantly higher in the DA group than in the SD group at 6 months. Recent real-world evidence has suggested that SLIT may confer FEV₁ benefits independent of the allergen type, though data specifically in non-asthmatic AR children remain scarce (37). In this context, our finding adds preliminary evidence to this underexplored area. Lombardi et al. emphasized that early AIT initiation during the AR stage, rather than as an add-on after medication failure, may help delay progression to asthma—a notion supported by large real-world datasets (38). The improvement in FEV₁ Z-score observed with dose escalation appears consistent with this early-intervention framework. To further explore the inflammatory basis of these changes, NO biomarkers (nNO and FeNO) were simultaneously monitored as indicators of upper and lower airway inflammation. Levels of both decreased significantly after treatment in both groups, suggesting a coordinated anti-inflammatory effect across the upper and lower airways. Mechanistically, Sahiner et al. highlighted that AIT-induced immune tolerance involves regulatory T cells, blocking antibodies (IgG4/IgA), and suppression of Th2-driven responses, which may underlie the coordinated biomarker changes observed in this study (39). Given the modest sample size of this exploratory substudy, these biomarker findings should be interpreted as hypothesis-generating signals warranting validation in larger, mechanistically focused trials.

This study has several limitations. First, we employed a standard-dose group as the comparator rather than a placebo, primarily for ethical reasons. While this design allows us to assess the incremental benefit of dose escalation, it may limit the independent assessment of absolute efficacy. Second, the 6-month follow-up period only allows for short-term efficacy observation. Whether the benefits of the higher-dose regimen can be sustained and translate into durable immune tolerance requires validation through longer studies, ideally covering the standard 3-year course of SLIT. Third, non-randomized group allocation, while reflective of clinical decision-making, introduces potential selection bias that statistical adjustment cannot fully eliminate. Fourth, the sample size of the subgroup undergoing pulmonary function, FeNO, and nNO testing was relatively small and may not be fully representative of the entire cohort, which limits the generalizability of these exploratory findings and may have reduced statistical power for some indicators. Additionally, we acknowledge that the RQLQ's validation age range (6–12 years) does not fully cover our entire cohort, which is a limitation of this study. Despite these limitations, the convergence of subjective symptom improvement, objective NAC-confirmed reduction in nasal reactivity, and significant improvement in FEV₁ Z-score collectively suggests that tolerance-guided individualized SLIT dose adjustment was associated with superior outcomes in symptom control, nasal sensitivity, and pulmonary function in this cohort. Randomized controlled trials with larger sample sizes and extended follow-up are warranted to confirm the long-term benefits and safety of individualized SLIT dose adjustment.

## 5 Conclusion

In this prospective cohort study of children with house dust mite AR, an individualized SLIT maintenance dose escalation strategy guided by tolerance was associated with greater alleviation of symptoms, reduced nasal allergen sensitivity, and additional lower airway function improvements within 6 months when compared with the conventional regimen. Based on subjective symptom scores as the primary assessment criterion, the standardized nasal allergen challenge can serve as an auxiliary objective tool, providing supplementary evidence for the efficacy evaluation of the individualized dose-adjustment strategy from the perspective of target organ reactivity. Furthermore, SLIT can coordinately reduce inflammatory levels in both the upper and lower airways. These findings suggest short-term clinical benefits of this individualized approach in this cohort. Given the observational design, randomized controlled trials are warranted to confirm the durability and generalizability of these effects.

## Data Availability

The original contributions presented in the study are included in the article/Supplementary Material, further inquiries can be directed to the corresponding author.
